# Heart Mitochondrial Metabolic Flexibility and Redox Status Are Improved by Donkey and Human Milk Intake

**DOI:** 10.3390/antiox10111807

**Published:** 2021-11-13

**Authors:** Giovanna Trinchese, Fabiano Cimmino, Gina Cavaliere, Luigi Rosati, Angela Catapano, Daniela Sorriento, Elisabetta Murru, Luca Bernardo, Luciana Pagani, Paolo Bergamo, Rosaria Scudiero, Guido Iaccarino, Luigi Greco, Sebastiano Banni, Marianna Crispino, Maria Pina Mollica

**Affiliations:** 1 Department of Biology, University of Naples Federico II, 80126 Naples, Italy; giovanna.trinchese@unina.it (G.T.); fabiano.cimmino@unina.it (F.C.); gina.cavaliere@unina.it (G.C.); luigi.rosati@unina.it (L.R.); angela.catapano@me.com (A.C.); rosaria.scudiero@unina.it (R.S.); crispino@unina.it (M.C.); 2 BAT Centre—Interuniversity Centre for Studies on Bioinspired Agro-Environmental Technology, University of Naples Federico II, 80055 Naples, Italy; 3 Department of Pharmacy, University of Naples Federico II, 80131 Naples, Italy; 4 Department of Advanced Biomedical Sciences, University of Naples Federico II, 80131 Naples, Italy; danisor@libero.it (D.S.); guido.iaccarino@unina.it (G.I.); 5 Department of Biomedical Sciences, University of Cagliari, 09042 Cagliari, Italy; m.elisabetta.murru@gmail.com (E.M.); banni@unica.it (S.B.); 6 Department of Childhood and Developmental Medicine, ASST Fatebenefratelli-Sacco, 20157 Milan, Italy; luca.bernardo@fbf.milano.it (L.B.); lucianapagani@gmail.com (L.P.); 7 Institute of Bioscience and Bioresources CNR, IBBR-UOS, 80131 Naples, Italy; paolo.bergamo@ibbr.cnr.it; 8 Department of Translational Medical Sciences, Section of Pediatrics, University of Naples Federico II, 80131 Naples, Italy; luigi.greco@unina.it; 9 Task Force on Microbiome Studies, University of Naples Federico II, 80100 Naples, Italy

**Keywords:** heart mitochondria, oxidative stress, nutrition, milk, antioxidants, fatty acids

## Abstract

The biological mechanisms linking nutrition and antioxidants content of the diet with cardiovascular protection are subject of intense investigation. It has been demonstrated that dietary supplementation with cow, donkey or human milk, characterized by distinct nutritional properties, triggers significant differences in the metabolic and inflammatory status through the modulation of hepatic and skeletal muscle mitochondrial functions. Cardiac mitochondria play a key role for energy-demanding heart functions, and their disfunctions is leading to pathologies. Indeed, an altered heart mitochondrial function and the consequent increased reactive oxygen species (ROS) production and inflammatory state, is linked to several cardiac diseases such as hypertension and heart failure. In this work it was investigated the impact of the milk consumption on heart mitochondrial functions, inflammation and oxidative stress. In addition, it was underlined the crosstalk between mitochondrial metabolic flexibility, lipid storage and redox status as control mechanisms for the maintenance of cardiovascular health.

## 1. Introduction

Human health depends on the maintenance of metabolic homeostasis to prevent the onset of chronic low-grade inflammation that causes later chronic disease, such as obesity and its comorbidities, some cancers and cardiovascular diseases (CVD) [1]. To maintain metabolic homeostasis the organism developed a “metabolic flexibility”, i.e., the capacity to efficiently adapt fuel oxidation to nutrients availability, managing sensing, uptake, trafficking, storage and expenditure of the nutrients [2]. The mitochondria, the most important players in the management of metabolic flexibility, adjust the capacity and efficiency of adenosine triphosphate (ATP) generation in response to energetic demands to keep the organism healthy [3]. Accordingly, mitochondrial dysfunction is linked to the development of metabolic pathologies [4]. 

The heart, along with the liver and skeletal muscle, is one of the metabolically very active organs since it needs intense energy production to generate the contractile force. It has been known for several decades that cellular ATP production plays a key role in heart function [5,6,7,8,9,10]. Accordingly, the expression of ATP synthase is linked to the function of the myocardium [11,12,13,14]. Cardiac mitochondria, beyond the regulation of energy metabolism, are known to regulate other essential cardiomyocyte functions like contractility, reactive oxygen species (ROS) production, apoptosis, as well as differentiation and development [15,16,17]. In addition, the evidence showing the contribution of cardiac mitochondria to the onset of several heart diseases, suggests their key role in the heart physiopathology [17,18,19]. Therefore, it is intriguing to hypothesize a nutritional intervention during early postnatal life to prevent CVD, acting on metabolic, microbiological and immunological development [20,21]. From this point of view, breastfeeding may have protective effects on CVD risk factor profiles in adulthood [22,23], leading to small reductions in adolescent and adult blood pressure levels [24,25,26], decreased total cholesterol and low-density lipoprotein (LDL) cholesterol levels in adulthood [25,27,28] and modest decrease in adult body mass index [29]. Nutritional intervention may imply the modulation of mitochondrial metabolism since it has been already demonstrated that acting on mitochondrial metabolism in high-metabolic-rate organs (i.e., liver and skeletal muscle), is an effective preventive strategy to relieve the burden of non-communicable diseases [30,31,32,33]. Nonetheless, the biological mechanisms underlying the modulatory activity of nutrition on the cardiovascular long-term protection are still far from being completely understood.

Recently, the attention of nutrition research switches from individual nutrients to a complete dietary pattern [34]. Accordingly, dietary recommendations specifically identified low-fat and fat-free dairy foods as components of healthy eating patterns.

Milk is undoubtedly an omnipresent food in the human diet: not only it is a gold standard food for infant nutrition, but humans keep drinking milk after weaning reinforcing its role as an important macro and micronutrients source. 

The main concern related to a possible influence of milk consumption on heart disease is related to the quality of its fat content, micronutrients components and, consequently, antioxidant properties [35,36]. The different physicochemical and nutritional properties among cow (CM), donkey (DM) or human (HM) milk may be responsible for their significant differences in the regulation of energy balance, glucose and lipid metabolism, inflammatory and redox state attributable to the modulation of hepatic and muscular mitochondrial function [37,38]. This evidence suggests that the potential influence of milk consumption in the regulation of cardiovascular health could be sought in the multiplicities of cardiac mitochondrial functions which extend beyond energy provision, such as ROS production and regulation of inflammatory responses. Therefore, in this study we investigate the effects of supplementation of rat diet with CM, DM and HM on heart mitochondrial functions, inflammation and redox homeostasis.

## 2. Materials and Methods

### 2.1. Materials Used

All chemicals were purchased from Sigma–Aldrich (St. Louis, MO, USA), unless specified otherwise. Cow milk (CM) was obtained from the “Nuova Latte Soc. Coop. Agr. A R.L.” (Contrada Isca SNC, Eboli, SA, Italy). Donkey milk (from the Ragusana breed) (DM) was obtained from the “Az. Agric. Garofalo Patrizia” (Contrada Valle Cerasa, Casalbordino, CH, Italy). Human milk (HM) was kindly provided by the milk bank of the Macedonio Melloni Hospital (Department of Childhood and Evolutionary Age Medicine, Milano, Italy). Standard rodent diet (4RF21) was purchased from Mucedola (Mucedola srl, Settimo Milanese, MI, Italy).

### 2.2. Animal Treatment and Energy Balance Assessment

Male Wistar rats (60 days old; 345 ± 7 g; Charles River, Calco, Lecco, Italy) were individually caged in a temperature-controlled room and exposed to a daily light–dark cycle (12/12 h) with free access to standard rodent diet (15.88 kJ/g) and drinking water. The rats were divided into four experimental groups (*n* = 7). Three of them received a 4-weeks supplementation with equicaloric intake (82 kJ) of raw CM, DM or HM drinking 21, 48 or 22 mL/day respectively, according to the experimental plan previously reported [37,38]. The last group did not receive milk supplement and was used as control. Despite the different volumes used, the energy density provided by the different milk supplements was kept the same. During the treatments, the body weights and food intake were monitored daily to calculate weight gain and gross energy intake. Spilled food and feces were collected daily for precise food intake and metabolizable energy intake calculation.

At the end of the treatments, the animals were anesthetized by intraperitoneal injection of chloral hydrate (40 mg/100 g body weight), and blood was taken from the inferior cava. Hearts were removed, parts of the hearts were washed in calcium-free PBS to remove blood residues, fixed for 24 h in 4% buffered paraformaldehyde, pH 7.4 and then embedded in paraffin. Other aliquots not immediately used for mitochondrial preparation and for histological analyses were stored at −80 °C. All experiments were conducted in compliance with national guidelines for the care and use of research animals and were approved by the institutional committee (Comitato Etico-Scientifico per la Sperimentazione Animale of the University of Naples Federico II) and authorized by the Italian Ministry of Health (Ethical approval number: 97/2019-PR).

Energy balance assessments were conducted over the 4 weeks of treatment by the comparative carcass evaluation [39]. Metabolizable energy intake was obtained by subtracting the energy measured in the feces and urine from the gross energy intake, which was determined from the daily food consumption and gross energy density. The gross energy density for the standard diet, CM, DM or HM (15.8, 14.04, 13.79 or 14.01 kJ/g, respectively) as well as the energy density of the feces and the carcasses were determined by bomb calorimeter (Parr adiabatic calorimeter, Parr Instrument Co., Moline, IL, USA). The evaluation of the energy, fat and protein content in animal carcasses was conducted according to a previously published protocol [39,40].

### 2.3. Evaluation of Inflammatory Markers

Commercially available ELISA kits were used to determine interleukin-1α (IL-1α), interleukin-10 (IL-10) and tumor necrosis factor-alpha (TNF-α) (Thermo Scientific, Rockford, IL; Biovendor R and D, Brno, Czech Republic) concentrations in serum and inheart homogenates.

### 2.4. Heart Lipid Content Measurement

Total lipids were extracted by the method of Folch et al. [41]. Total lipid quantification was performed by the method of Chiang et al. [42]. Aliquots of total lipid extract from heart were mildly saponified as previously described [43] in order to obtain free fatty acids. Separation of unsaturated fatty acids was carried out with an Agilent 1100 HPLC system (Agilent, Palo Alto, CA, USA) equipped with a diode array detector [44]. Saturated fatty acids were measured as fatty acid methyl esters, by a gas chromatograph (Agilent, Model 6890, Palo Alto, CA, USA) equipped with a flame ionization detector; the split ratio was set at 20:1 and the injection port temperature was 270 °C and a 100 m HP-88 fused capillary column (Agilent, Palo Alto, CA, USA) were used. Data were acquired by the Agilent OpenLab ChemStation software system (CDS 2.4, Agilent Technologies, Santa Clara, CA, USA).

Quantification of endocannabinoidome was conducted by an Agilent 1100 HPLC system equipped with a mass spectrometry Agilent Technologies QQQ triple quadrupole 6420 with electrospray ionization source (Agilent Technologies, Santa Clara, CA, USA), using positive mode (ESI+) and using as internal standards their deuterated homologs as previously described [45]. Data were acquired by the MassHunter workstation acquisition software (version B.08.02, Agilent Technologies, Santa Clara, CA, USA), analyzed with MassHunter software for qualitative (version B.08.00 SP1 Agilent Technologies, Santa Clara, CA, USA) and quantitative analyses (version B.09.00, Agilent Technologies, Santa Clara, CA, USA). Endocannabinoidome compounds were expressed as pmol/g tissue.

### 2.5. Mitochondrial Isolation Procedure 

The hearts were finely minced and washed in a medium containing 100 mmol/LKCl, 50 mmol/L Tris, pH 7.5, 5 mmol/L MgCl_2_, 1 mmol/L ethylenediamine tetraacetic acid (EDTA), 5 mmol/L ethylene glycol tetraacetic acid (EGTA), 1 g/L fatty acid–free bovine serum albumin (BSA). Tissue fragments were treated with protease nagarse (E.C. 3.4.21.62; 1 mg/g tissue) for 5 min, washed, homogenized for 1 min with the above medium (1:8, *w*/*v*) in a Potter Elvehjem homogenizer (Heidolph, Kelheim, Germany) set at 500 rpm (4 strokes/min) and filtered. The homogenate was centrifuged at 3000× *g* for 10 min, the pellet containing unbroken cells, nuclei, as well as mitochondria, was washed twice, resuspended in a suspension medium containing 250 mmol/L sucrose, 50 mmol/L Tris, pH 7.5, 1 g/L fatty acid–free BSA and centrifuged at 700× *g* for 10 min. The resulting supernatant fraction was again centrifuged at 3000× *g* for 10 min. The mitochondrial pellet was washed twice and finally re-suspended in the suspension medium. The protein content of the mitochondrial suspension was determined by the method of Hartree [46] using BSA as the protein standard. Isolated mitochondria were then used for the determination of respiratory parameters. 

### 2.6. Measurement of Mitochondrial Oxygen Consumption

Mitochondrial oxygen consumption was polarographically measured by a Clark-type electrode (Yellow Springs Instruments, Yellow Springs, OH, USA) at 30 °C. In detail, isolated mitochondria (0.5 mg protein/mL) were incubated in a medium containing 30 mM KCl, 6 mM MgCl_2_, 75 mM sucrose, 1 mM EDTA, 20 mM KH_2_PO_4_ pH 7.0, and 0.1% (*w*/*v*) fatty acid-free BSA. State 3 oxygen consumption was measured in the presence of 10 mM succinate + 3.75 mM rotenone or of 2.5 mM pyruvate + 5 mM malate and after the addition of 0.6 mM adenosine diphosphate (ADP). State 4 was obtained in the absence of ADP. The rate of mitochondrial fatty acid oxidation was assessed in the presence of 2.5 mM malate + 40µM palmitoyl-L-carnitine and 0.6 mM of ADP. The respiratory control ratio (RCR) was calculated as the ratio between states 3 and 4. In control experiments, we assured the quality of our mitochondrial preparation by checking that contamination of mitochondria by other ATPase-containing membranes was lower than 10%, and the addition of cytochrome c (3 nmol/mg protein) only enhanced state 3 respiratory rate by approximately 10%.

### 2.7. Evaluation of Redox Status

The rate of mitochondrial hydrogen peroxide (H_2_O_2_) release was assayed by following the linear increase in fluorescence (excitation 312, emission 420 nm) due to the oxidation of homovanillic acid in the presence of horseradish peroxidase [47]. Superoxide dismutase (SOD) specific activity was measured spectrophotometrically (550 nm) at 25 °C, by monitoring the decrease in the reduction rate of cytochrome c by superoxide radicals, generated by the xanthine–xanthine oxidase system [48].

Catalase (CAT) activity was measured in heart homogenates, by monitoring the decrease in absorbance at 240 nm due to the decomposition of H_2_O_2_ [49].

To determine the lipid peroxidation in heart homogenate, the level of malondialdehyde (MDA) was measured using the thiobarbituric acid reaction (TBAR) method. MDA reacts with thiobarbituric acid (TBA) to form a pink chromogen that is detected at the wavelength of 532 nm. MDA values were expressed as nanomoles per milligram of protein [50].

Reduced glutathione (GSH) and oxidized glutathione (GSSG) concentrations in the hearts of differently treated rats were measured with the dithionitrobenzoic acid-GSSG reductase recycling assay [51]. Upon normalization to the protein content, GSH and GSSG amount were finally expressed as nmoles/mg prot/min.

### 2.8. Real-Time PCR

Total RNA was extracted from 50 mg of cardiac tissues (*n* = 3/group) using a TRIzol reagent (Invitrogen, (ThermoFisher), Waltham, MA, USA), and cDNA was synthesized using the ThermoScript RT-PCR System (Invitrogen, (ThermoFisher), Waltham, MA, USA), following the manufacturer instruction. After reverse transcription reaction, real-time quantitative polymerase chain reaction (RT-PCR) was performed with the SYBR Green real-time PCR master mix kit (Applied Biosystems, Foster City, CA, USA) as described. The reaction was visualized by SYBR Green-derived fluorescence analysis on the StepOne instrument (Applied Biosystem, Applied Biosystem, (ThermoFisher) Waltham, MA, USA). All standards and samples were assayed in triplicate. All values obtained were normalized to the endogenous control 18S using the formula 2^−ΔCt^ and results are expressed as relative quantification (RQ). Primer sequences are shown in Table 1.

### 2.9. Light Microscopy

Serial 6 µm sections of the cardiac tissue embedded in paraffin were processed for routine histological analysis and stained with Mallory’s trichromic stain, as previously described [52]. Briefly, the staining was carried out in two steps. In the first step, the sections were stained in 1% acid fucsin solution in distilled water; the second step involved counterstaining in a Mallory solution with 0.5% methyl blue, 2% orange G, and 2% oxalic acid in distilled water. Images were acquired with a camera connected to an IBM computer running the Kontron Elektronik KS 300 image analysis system (Carl Zeiss MicroImaging, Milan, Italy).

### 2.10. Statistical Analyses

All data were presented as means ± SEM. Differences among groups were compared by one-way ANOVA followed by the Bonferroni post hoc test to correct for multiple comparisons. Fatty acids and endocannabinoidome data were not normally distributed; therefore, the differences between the groups were assessed using nonparametric Kruskal–Wallis test (one-way ANOVA on ranks) followed by Dunn’s correction for multiple comparisons. Data were analyzed using GraphPad Prism 6.0 (GraphPad Software Inc., La Jolla, CA, USA) with *p* ≤ 0.05 as the cut-off for statistical significance between groups. Data with different superscript letters were significantly different according to the statistical analysis, as specified in the figures and tables.

## 3. Results

### 3.1. Distinct Milk Administration Differently Modulates Body Weight Gain, Composition and Efficiency

**Figure 1:**
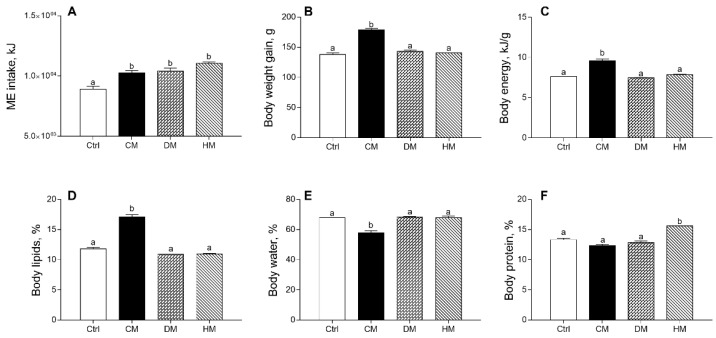
Cow milk (CM), donkey milk (DM) or human milk (HM) diet supplementation influences body weight gain and composition in rats. Metabolizable energy intake (ME) (**A**), body weight gain (**B**) during the study period (4 weeks), body energy (**C**), lipids (**D**), water (**E**) and protein (**F**) were reported. Data were expressed as the means ± SEM *n* = 7 animals/group. Different letters (a,b) on top of the bars indicate statistically significant differences (*p* < 0.05) among groups.

The effects produced in rats by diet supplementation of milk from three distinct animal species on energy balance and body composition are shown in Figure 1, confirming previous data [37]. Equicaloric administration of different milk provided similar metabolizable energy intake in all treated rats, significantly higher than controls (*p* < 0.001) (Figure 1A). Body weight gain, body energy and lipids percentage in CM group were significantly higher than in the control group (*p* < 0.001) (Figure 1B–D), while a decrease in body water was observed in CM-fed animals as compared to other groups (*p* < 0.001) (Figure 1E). Body protein content (%) was significantly increased by HM treatment (*p* < 0.001) (Figure 1F).

### 3.2. Modulation of Serum and Tissue Inflammatory Profile in Rats Fed with Different Milk

**Figure 2:**
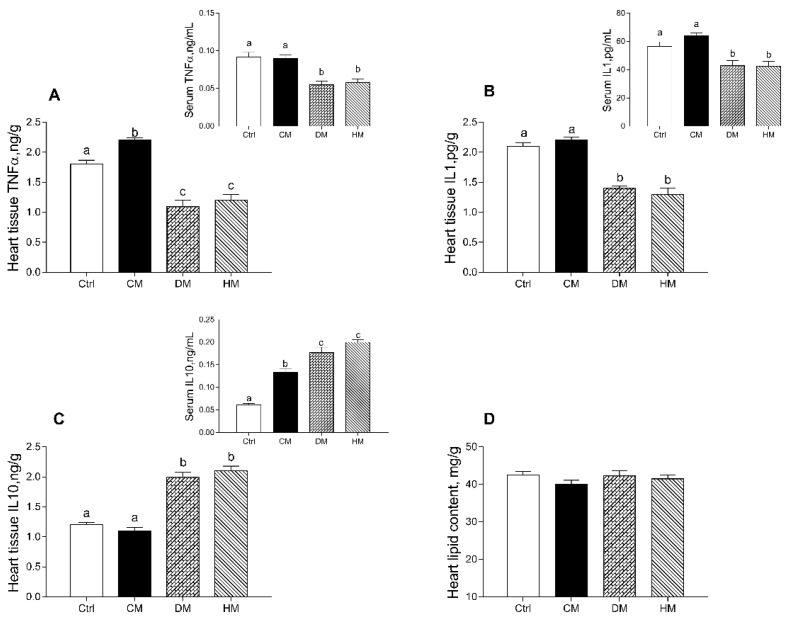
Cow milk (CM), donkey milk (DM) or human milk (HM) diet supplementation differently modulates inflammation in rat serum and cardiac tissue. Proinflammatory cytokines, such as tumor necrosis factor (TNF-α) (**A**) and interleukin-1 (IL-1) (**B**) and anti-inflammatory marker interleukin-10 (IL-10) (**C**) were measured in serum and heart (upper panels). Total lipid content (**D**) was determined in heart tissue. Data were expressed as the means ± SEM *n* = 7 animals/group. Different letters (a,b,c) on top of the bars indicate statistically significant differences (*p* < 0.05) among groups.

In rats fed with DM and HM, compared to other groups, the levels of proinflammatory markers (TNF-α and IL-1α) were significantly reduced in both serum (*p* < 0.01) and cardiac tissue (*p* < 0.001) (Figure 2A,B). In addition, the levels of TNF-α in tissue of CM-treated rats were also higher than control animals (*p* < 0.05) (Figure 2A). Moreover, increased levels of the anti-inflammatory cytokine IL-10 were observed in DM- and HM-treated animals both in serum (*p* < 0.01) and in the heart (*p* < 0.001) (Figure 2C). In cardiac tissue, no significant differences in total lipid content were observed in all treated groups (Figure 2D).

### 3.3. Heart Mitochondrial Oxidative Capacity Is Modulated by the Administration of Milk from Different Animal Species

**Figure 3:**
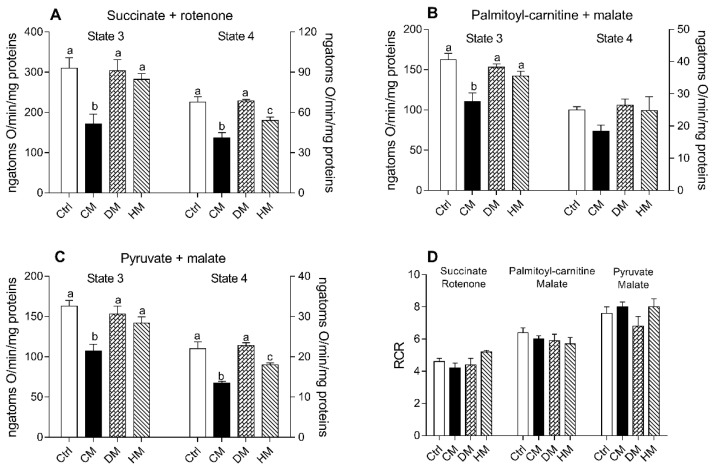
Cow milk (CM), donkey milk (DM) or human milk (HM) diet supplementation modulates the rat cardiac mitochondrial oxidative capacity. The mitochondrial respiration rates were evaluated in the presence of succinate-rotenone (**A**) or palmitoyl-carnitine-malate (**B**) or pyruvate-malate (**C**) as substrates, in the presence (state 3) or absence (state 4) of ADP. Relative respiratory control ratios (RCR) were reported (**D**). Data were expressed as the means ± SEM n = 7 animals/group. Different letters (a,b,c) on top of the bars indicate statistically significant differences (*p* < 0.05) among groups.

Respiration rates of cardiac mitochondria were measured in the presence of succinate-rotenone, palmitoyl carnitine-malate or pyruvate-malate as substrates. It is noteworthy that the stoichiometry of ATP synthesis to oxygen consumption is lower when lipids rather than carbohydrates are oxidized [53]. Regardless of the substrates used, state 3 and state 4 mitochondrial oxygen consumptions were significantly reduced in CM-treated animals when compared to the other groups (*p* < 0.01 vs control and DM-treated) (Figure 3A–C). In the presence of palmitoyl-carnitine, no significant differences were observed in state 4 respiration rates between different groups (Figure 3B). The quality of mitochondrial preparations was assessed by the respiratory control ratio (RCR) (Figure 3D).

### 3.4. Cardiac Redox Status Is Modulated by Different Milk Administration in Rats

**Figure 4:**
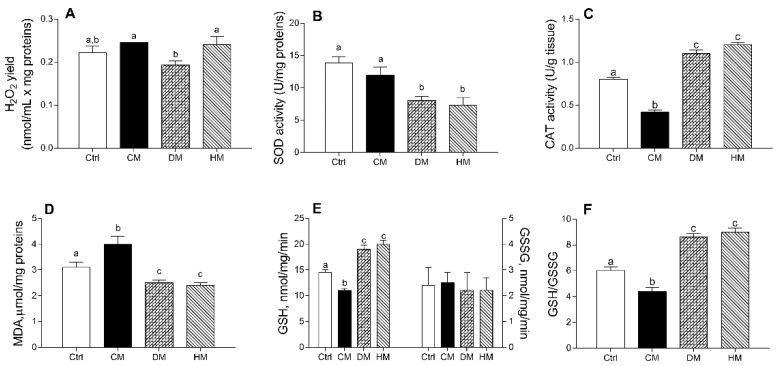
Cow milk (CM), donkey milk (DM) or human milk (HM) diet supplementation exhibits different efficacy in modulating rat cardiac mitochondrial ROS production, enzymatic antioxidant defenses, lipid peroxidation and cellular GSH/GSSG ratio. Mitochondrial H_2_O_2_ yield (**A**), superoxide dismutase (SOD) (**B**) and catalase (CAT) activities (**C**) were reported. Lipid peroxidation index (MDA) (**D**), GSH and GSSG levels (**E**,**F**) were determined in heart homogenates. Data were expressed as the means ± SEM n = 7 animals/group. Different letters (a,b,c) on top of the bars indicate statistically significant differences (*p* < 0.05) among groups.

The DM administration decreased mitochondrial H_2_O_2_ release compared to CM or HM treatments (*p* < 0.05) (Figure 4A). DM- and HM-treated groups showed a significantly lower SOD activity than control (*p* < 0.01) or CM-treated animals (*p* < 0.05) (Figure 4B). In addition, CM-supplemented rats showed a significant reduction in CAT activity as compared to other groups (*p* < 0.001) (Figure 4C) and exhibited the highest lipid peroxidation index (MDA) (*p* < 0.01 vs control; *p* < 0.001 vs. DM- and HM), whereas the lowest values were detected in DM- and HM-animals (Figure 4D).

Diet supplementation with DM or HM improved the antioxidant state. Indeed, while no differences in GGSG content was observed between different groups, GSH levels significantly decreased in CM- (*p* < 0.01) and increased in DM- or HM-treated animals (*p* < 0.001) when compared to controls (Figure 4E), resulting in the lowest GSH/GSSG ratio in CM-group (*p* < 0.01 vs. control) and in the highest value in the DM- and HM-treated groups (*p* < 0.001) (Figure 4F).

### 3.5. Distinct Milk Administration Differently Modulates Cardiac Expression of the Mitochondrial Respiratory Chain Complexes Genes

**Figure 5:**
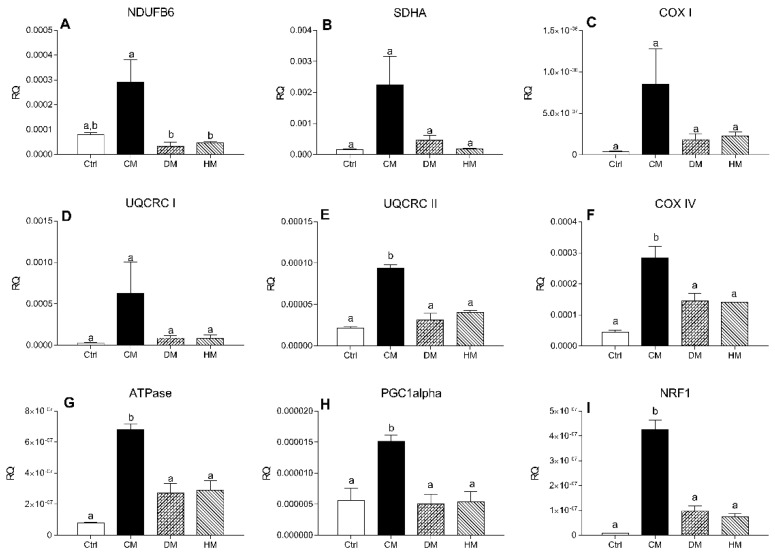
mRNA levels of complex I-II subunits in heart tissue. The transcription levels of *NDUFB6* (**A**), *SDHA* (**B**), *COX-I* (**C**), *UQCRC-I* (**D**), *UQCRC-II* (**E**), *COX-IV* (**F**), *ATPase* (**G**), *PGC-1alpha* (**H**) and *NRF* (**I**) genes were evaluated by Real-Time PCR. Results are expressed as relative quantification (RQ) and are the mean of three independent experiments (*n* = 3/group). Different letters (a,b) on top of the bars indicate statistically significant differences (*p* < 0.05) among groups.

To investigate the effect of milk diet supplementation on expression levels of genes encoding subunits of mitochondrial respiratory chain complex, the transcript levels of two subunits of the complex I-II and of five subunits of complex III-IV were evaluated by RT-PCR. mRNA levels of *NDUFB6* and *SDHA*, components of complexes I and II respectively, *COX I* and *UQCRC I* did not change in the treated groups compared with controls, although a non-significant increase was observed in CM treated rats (Figure 5A–D). The expression levels of *UQCRC II*, *COX IV*, *ATPase* (*p* < 0.001, CM vs. control) (Figure 5E–G) and regulators of mitochondrial biogenesis *PGC-1alpha* (*p* < 0.05) and *NRF1* (*p* < 0.001) (Figure 5H,I) were significantly increased in rats treated with CM compared with other groups.

### 3.6. Distinct Milk Administration Affects the Histological Features of the Heart Muscle Fibers

**Figure 6:**
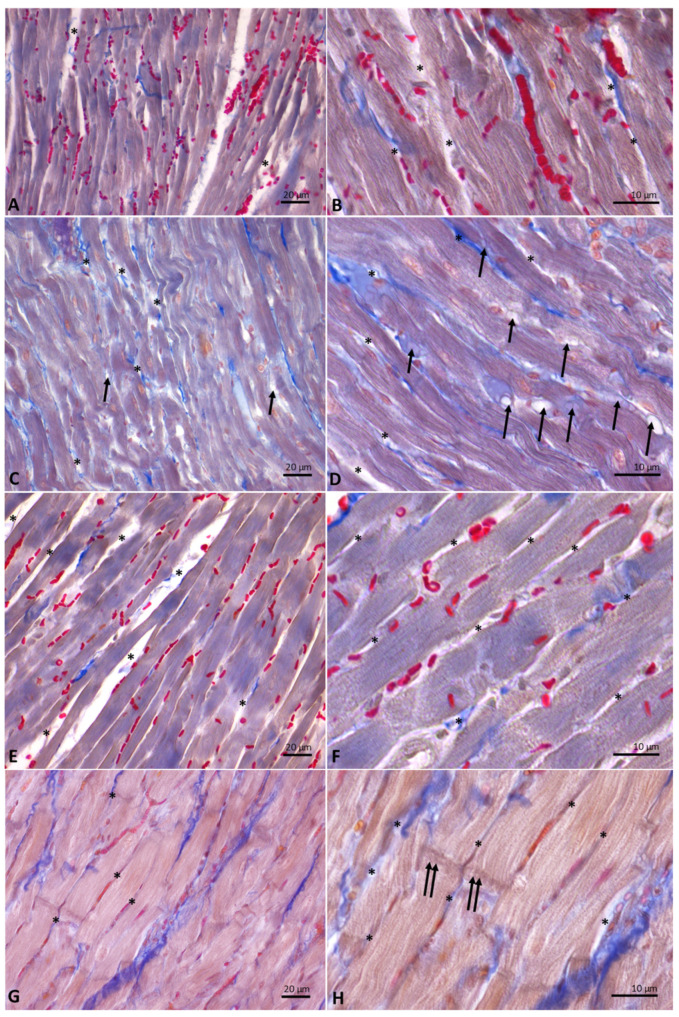
Longitudinal sections of rat hearts stained with Mallory’s trichrome stain. (**A**,**B**): controls. (**C**,**D**): donkey milk (DM) diet supplementation. (**E**,**F**): human milk (HM) diet supplementation. (**G**,**H**): cow milk (CM) diet supplementation. Legends: arrow, adipocyte; asterisk, intercellular space, double arrow, intercalated discs. Scale bar: (**A**,**C**,**E**,**G**), 20 µm; (**B**,**D**,**F**,**H**), 10 µm.

Cardiac fibers of control (Figure 6A,B) and DM- (Figure 6C,D) and HM-treated animals (Figure 6E,F) showed normal morphology. Specifically, from the analysis of Mallory trichrome stained longitudinal sections of hearts emerged the presence, in these experimental groups, of regular-shaped cardiomyocytes, in which the transverse streaks and regular intercellular spaces are evident; in the intercellular spaces, collagen fibers appear blue-stained. In the sections of animals supplemented with DM, the presence in the intercellular spaces of cells with the typical adipocyte shape is also evident (Figure 6D). On the contrary, heart sections from animals supplemented with CM (Figure 6G,H) showed hypertrophic cardiomyocytes, in which transverse banding and intercalated discs are detectable. In these animals, due to the increase in the volume of cardiomyocytes, the intercellular spaces appear strongly reduced.

### 3.7. Distinct Milk Administration Modifies Lipid Profile and Endocannabinoidome in the Cardiac Tissue

Fatty acid profile shows a general higher incorporation of n-3 highly polyunsaturated fatty acids (HPUFAs), and in particular docosahexaenoic acid (DHA), in hearts of rats treated with DM and HM (Table 2), while *N*-acylethanolamines (NAE) profile shows a significant decrease of *N*-arachidonoylethanolamide (AEA) and *N*-docosahexaenoylethanolamide (DHEA) in the same groups (Table 3).

## 4. Discussion

The investigation of the biological mechanisms linking nutrition, antioxidants properties and cardiovascular protection continues to be a great challenge. Our data demonstrated, for the first time, that the administration of milk from different animal species in rat model system, differently modulates heart lipid metabolism, inflammation and oxidative state by affecting the regulation of the mitochondrial function. These results are particularly relevant since mitochondria play a crucial role in cardiac energy output and are critically important in energy-demanding cardiac functions [54]. It is noteworthy that the role of mitochondria is not limited to bioenergetic regulation, but it extends to ion homeostasis, apoptosis and synthesis of key molecules related to inflammation and redox state [55]. The heart, besides liver and skeletal muscle, is one of the most metabolically active organs in the body since it needs intense energy production to generate the contractile force. Therefore, cardiac mitochondria represent the key organelles for the maintenance of cardiac health, and the control of mitochondrial function is a crucial step in the prevention/therapy of cardiac diseases [56].

We previously demonstrated that the diet supplementation of CM, DM or HM in rats, differently regulated metabolic and inflammatory parameters attributable to the modulation of hepatic and muscular mitochondrial function [37,38]. These data validated the alteration of mitochondrial function as part of the wide spectrum of metabolic changes induced by nutrition-dependent low-grade inflammation [57]. In particular, DM and HM are able to improve the use of fat as metabolic fuel for hepatic and skeletal muscle mitochondria, decreasing the accumulation of body lipids [37,38]. Thus, DM and HM, compared to CM, yield similar metabolizable energy, but increased energy expenditure and consequent decrease in body weight and body lipid gain [37]. We hypothesized that the metabolic effects of HM and DM depend, at least in part, on their higher content in PUFA omega-3, which are known to be involved in energy balance, lipid metabolism and prooxidant status, through the modulation of mitochondrial functions [30]. On the other hand, the increased inflammatory state and oxidative stress of CM-treated rats, may be related to the high concentrations of saturated fatty acids in CM [58]. However, we cannot exclude that the increased body weight gain of CM animals can contribute to their metabolic state. Here, we confirmed the influence of milk supplementation from different animal species in the cytokine’s levels of rat serum and heart tissue, showing that DM and HM administration decreases TNF-α and IL-1 in both serum and heart, and increases IL-10, an anti-inflammatory mediator. TNFα and IL-1β, together with the reactive nitrogen intermediate nitric oxide, may induce mitochondrial damage [59,60]. Moreover, TNFα and IL-1β decrease the activity of mitochondrial respiratory complex I, ATP production and mitochondrial membrane potential. These mediators also induce the accumulation of significant amounts of ROS [61,62]. Therefore, mitochondrial dysfunctions and inflammation are tightly interlinked processes promoting a vicious inflammatory cycle and increased oxidative stress [63].

By analyzing the modulation of heart mitochondrial function following the administration of milk from different animal species, we observed that CM-fed rats exhibited decreased respiratory capacity and decreased fatty acid oxidation rate as compared to other groups. Indeed, state 3 respiration significantly decreased both in presence of NADH-linked (pyruvate) and FADH-linked (succinate) substrate. A decrease in succinate State 3 respiration may be due to defects in the activity of substrate oxidation reactions (complex II, complex III, complex IV and dicarboxylate carrier) and/or in the activity of the phosphorylation reactions (ANT, ATP synthase and phosphate carrier). However, the gene expression analysis conducted in milk-treated rats suggested that the observed mitochondrial alteration in CM-fed animals could be compensated by upregulation of genes related to mitochondrial biogenesis (*PGC-1alpha* and *NRF1*) and an increase of the transcript levels of the mitochondrial subunits of the respiratory chain complexes. The role of PGC-1alpha in the control of mitochondrial number and function in cardiac tissue is still controversial [64]. Some studies have suggested that overexpression of PGC-1alpha may be beneficial for the heart under pressure load [65]. However, the overexpression of PGC-1alpha may not improve mitochondrial function and myocardial contractile function [66]. The animals of CM-fed group showed a decreased fatty acid oxidation rate and hypertrophic cardiomyocytes, although no significant differences were found in cardiac lipid content among differently treated groups. These results are in line with previous study demonstrating that cardiac hypertrophy results in a downregulation of fatty acid oxidation enzyme expression and reduced capacity for mitochondrial oxidation of fats [67]. Therefore, it can be hypothesized that PGC-1alpha overexpression is aimed at rescuing the decreased mitochondrial oxidative capacity in CM group.

These results confirm that cardiomyocytes develop well-coordinated quality control mechanisms that maintain overall mitochondrial health. Cardiac mitochondria are also the primary source of ROS, which contribute to mitochondrial dysfunction, cardiomyocyte damage and heart failure [68,69,70,71]. ROS production is typically controlled by intracellular and intramitochondrial scavenging systems. Pathological ROS levels in the heart typically occur when ROS production outpaces endogenous scavenging capacity, leading to damaged proteins and lipids, triggering cell-death cascades and evoking synchronized collapses in the cellular energy grid [72]. In this study, pathological levels of ROS were not found in any milk-treated group. However, our data showed that DM and HM administration decreased mitochondrial H_2_O_2_ release and increased catalase activity leading to higher GSH/GSSG ratio, although SOD activity decreased. Accordingly, in the same groups of animals it was observed a decreased lipid peroxidation, indicated by the reduced MDA levels. Conversely, the impairment in mitochondrial function observed in CM-fed rats was corroborated by the altered redox status as demonstrated by the lowest GSH/GSSG ratio and the reduction in catalase activity. The compromised oxidative state in CM group is mirrored in higher MDA levels in the heart of these animals. These increased levels of MDA are particularly relevant since the lipid peroxidation is the first step on the way to the development of CVD [73]. It is noteworthy that fatty acids accumulating in the vicinity of mitochondria are particularly vulnerable to ROS-induced lipid peroxidation, which has in turn toxic effects on mtDNA, RNA and proteins of the mitochondrial machinery, leading to mitochondrial dysfunction [74].

Fat cells were visualized in the intercellular spaces of some area of heart from animals fed with DM. Fatty acids are an important fuel source for heart and skeletal muscle, providing over 70% of the energy needs for cardiac functions [75]. In healthy subjects it was demonstrated that the aging, the short-term caloric restriction and starvation provoked a dose-dependent increase in myocardial triglyceride content and a decrease in diastolic function [76]. Cardiac and perivascular adipose tissue may act as a local energy supplier and/or as a buffer against toxic levels of free fatty acids in the myocardium and the arterial circulation [77]. Indeed, it has been hypothesized that it initially serves to compartmentalize the toxic lipid species, but subsequently promotes cardiac damage [76]. Thus, the cardiac and perivascular fat depots, in a healthy situation, exert a protective modulation of vascular function, but when they expand, become lipotoxic, prothrombotic and proinflammatory organ. The adipose tissue, establishing intense crosstalk with cardiac tissue, may shift from being protective to being detrimental, depending on its residual substrate buffering capacity and inflammatory status. In addition, fat around blood vessels and heart may play a supportive role, not only from a mechanical point of view, but also as a vasocrine and paracrine source of cytokines increasing the release of the anti-inflammatory cytokine IL-10 and decreasing the release of IL-1 and TNFα, as we observed in DM-fed group.

Unexpectedly, analysis of the NAE showed reduction of *N*-docosahexaenoylethanolamide (DHEA) in rats fed DM, and reduction of *N*-arachidonoylethanolamide (AEA) in rats fed DM or HM, despite arachidonic acid, precursor for AEA, did not change in both groups and DHA, precursor for DHEA, increased significantly. These data suggest that differences in AEA and DHEA might be ascribed to their degradation by fatty acid amide hydrolase (FAAH) [78]. In addition, we previously found that in skeletal muscle of rats fed DM and HM the levels of OEA increased [38], while no difference was observed in the heart of the same groups of animals. These results may also be explained by an increased FAAH activity in the heart [79]. It is noteworthy that AEA is a ligand of cannabinoid type 1 (CB1) and transient potential vanilloid 1 (TRPV1) receptors, and the role of CB1 and AEA in the heart is quite controversial [80]. Therefore, further studies are needed to evaluate whether and how these changes may influence heart function.

## 5. Conclusions

Our data demonstrated, for the first time, that the administration of cow, donkey or human milk differently modulates heart lipid metabolism, inflammation and oxidative state by affecting heart mitochondrial function. The different efficacy of various types of milk in modulating cardiac mitochondrial ROS production, enzymatic antioxidant defenses and lipid storage, utilization and peroxidation, underlines the functional role of different milk in metabolic and inflammatory homeostasis. This study strengthens the interlink between mitochondrial metabolic flexibility, lipid storage and redox status as control mechanisms for the maintenance of cardiovascular health.

## Figures and Tables

**Table 1:** Primer sequences for Real-Time PCR.

Gene	Accession n° (NCBI Database)	Sense Primer Sequence	Anti-Sense Primer Sequence
*18 S*	NR_003278	5’-GTAACCCGTTGAACCCCATT-3’	5’CCATCCAATCGGTAGTAGCG-3’
*NDUFB 6*	NM_001033305.3	5’-ATAACTTTTTGCGGGACGGG-3’	5’-CAGGAAAATCTCTCATTGGTG-3’
*SDHA*	NM_023281.1	5’-CATACTGTTGCAGCAGCACAGG-3’	5’-CCACCAAATGCACGCTGATA-3’
*ATPASE*	NM_001302213.1	5’-TGTGGAAGGAAGTGGGCAA-3’	5’-CCACTATGAGCTGGAGCCGT-3’
*COX 1*	NP_904330.1	5’-GAAGAGACAGTGTTTCATGTGGTGT-3’	5’-TCCTGGGCCTTTCAGGAATA-3’
*COX 4*	NM_001293559.1	5’-GAGCACATGGGAGTGTTGTG-3’	5’-CTGTCTTCCATTCATTGGTGCC-3’
*UQCRC I*	NM_025407.2	5’-CCTACGCACTCGAGAGCAC-3’	5’-AGGTGTGCCCTGGAATGCTG-3’
*UQCRC II*	NM_025899.2	5’-TCCCTCAAAGTTGCCCC-3’	5’-GCAAGACGTAGTAAATGTGAG-3’
*PGC1 ALPHA*	NM_008904.2	5’-AAACTTGCTAGCGGTCCTCA-3’	5’-TGGCTGGTGCCAGTAAGAG-3’
*NRF1*	NM_001164226.1	5’-GCACCTTTGGAGAATGTGGT-3’	5’-GGGTCATTTTGTCCACAGAGA-3’

*18 S*: 18 S Ribosomal RNA; *NDUFB6*: NADH-Ubiquinone Oxidoreductase Subunit B6; *SDHA*: succinate dehydrogenase complex flavoprotein subunit A; *COX*: cytochrome c oxidase 1 and 4; *UQCRC*: Ubiquinol-Cytochrome C Reductase Core Protein I and II; *PGC-1 alpha*: peroxisome proliferator-activated receptor gamma, coactivator 1 alpha; *NRF1*: nuclear respiratory factor 1.

**Table 2:** Changes of fatty acid profile in heart of rats fed with CM, DM or HM diet supplementation.

nmol/g Tissue	Control	CM-Treated	DM-Treated	HM-Treated
**ALA,18:3n3**	258.57 ± 21.35	235.50 ± 38.53	262.00 ± 26.20	238.72 ± 23.99
**EPA,20:5n3**	180.42 ± 12.61 ^a,b^	149.34 ± 12.61 ^a^	187.97 ± 24.62 ^b^	183.21 ± 6.77 ^a,b^
**DPA,22:5n3**	902.76 ± 62.55 ^a,b^	831.42 ± 90.42 ^a^	974.87 ± 86.88 ^a,b^	906.75 ± 42.24 ^b^
**DHA,22:6n3**	8938.36 ± 436.47 ^a^	9616.58 ± 158.38 ^a^	11,132.58 ± 336.50 ^b^	10,290.31 ± 295.78 ^a,b^
**LA,18:2n6**	21,911.79 ± 372.92 ^a,b,c^	18,689.10 ± 902.29 ^b,c^	23,642.50 ± 847.93 ^a^	19,785.46 ± 379.40 ^b^
**ETA,20:3n6**	632.89 ± 60.42	674.66 ± 32.68	653.45 ± 60.58	747.09 ± 41.46
**AA,20:4n6**	13,445.11 ± 404.61	13,201.11 ± 651.81	13,963.23 ± 1144.02	14,381.91 ± 573.23
**DPA,22:5n6**	604.56 ± 31.19 ^a^	693.92 ± 7.68 ^b^	655.69 ± 11.55 ^a,b^	676.23 ± 25.97 ^a,b^
**DTA,22:4n6**	756.65 ± 59.39	777.80 ± 58.09	761.79 ± 60.48	730.88 ± 35.32
**14:1 n7**	156.33 ± 10.34 ^a^	157.32 ± 3.01 ^a^	101.23 ± 1.50 ^b^	115.61 ± 8.00 ^a^
**POA,16:1n7**	647.98 ± 71.88	678.39 ± 189.96	546.32 ± 90.22	461.51 ± 70.58
**OA,18:1n9**	7238.21 ± 296.97	6939.20 ± 820.32	7438.93 ± 771.68	7596.36 ± 375.55
**18:1n7**	5054.95 ± 167.26	4401.26 ± 324.03	4313.26 ± 200.96	4433.04 ± 123.26
**8:0**	106.20 ± 17.43	136.47 ± 17.36	99.76 ± 17.58	141.32 ± 31.38
**10:0**	42.73 ± 9.50	68.62 ± 27.13	44.59 ± 11.27	45.39 ± 8.22
**12:0**	58.49 ± 20.13	106.85 ± 25.12	29.31 ± 3.36	107.30 ± 16.65
**MA,14:0**	218.29 ± 21.86 ^a^	467.59 ± 57.14 ^b^	228.89 ± 19.40 ^a,b^	251.69 ± 15.93 ^a,b^
**PA,16:0**	13,368.47 ± 549.03	12,908.57 ± 612.14	12,195.98 ± 505.56	12,508.22 ± 357.77
**17:0**	494.22 ± 49.96	418.46 ± 44.82	320.53 ± 17.76	297.69 ± 33.37
**SA,18:0**	14,044.16 ± 383.02	13,532.07 ± 387.28	13,013.23 ± 471.47	14,4424.90 ± 327.01

Data represent mean ± SEM for 7 animals per group and are expressed as nmoles/g tissue. Different superscripted letters indicate statistically significant differences (*p* < 0.05) (one-way ANOVA nonparametric measures with Kruskal–Wallis test). ALA, α-linolenic acid; EPA, eicosapentaenoic acid; DPA, docosapentaenoic acid; DHA, docosahexaenoic acid; DTA, docosatetraenoic acid; LA, linoleic acid; ETA, eicosatrienoic acid; AA, arachidonic acid; POA, palmitoleic acid; OA, oleic acid; MA, myristic acid; PA, palmitic acid; SA, stearic acid. Different letters (a,b,c) next to values indicate statistically significant differences (*p* < 0.05).

**Table 3:** Changes of endocannabinoidome profile in heart of rats fed with CM, DM or HM diet supplementation.

pmol/g Tissue	Control	CM-Treated	DM-Treated	HM-Treated
**POEA**	12.53 ± 2.69 ^a,b^	7.80 ± 0.52 ^b^	13.75 ± 1.13 ^a^	9.34 ± 1.00 ^a,b^
**AEA**	55.56 ± 3.55 ^a^	45.74 ± 3.55 ^a,b^	21.54 ± 1.84 ^c^	27.34 ± 0.36 ^b^
**DHEA**	126.03 ± 3.94 ^a^	132.81 ± 4.73 ^a^	78.59 ± 6.18 ^b^	97.92 ± 5.65 ^a,b^
**LEA**	100.97 ± 7.36	105.19 ± 6.25	108.78 ± 8.01	103.53 ± 10.09
**PEA**	140.32 ± 9.19	129.41 ± 11.78	125.08 ± 12.16	144.48 ± 17.80
**OEA**	169.75 ± 11.26	153.75 ± 7.07	169.28 ± 11.06	171.49 ± 13.82
**DTEA**	9.70 ± 1.20	11.44 ± 3.19	7.23 ± 0.73	9.05 ± 1.28
**SEA**	69.19 ± 4.08	68.43 ± 3.38	71.08 ± 5.83	69.19 ± 7.58
**2AG**	4251.89 ± 307.50	5076.72 ± 508.05	3446.42 ± 217.36	4662.21 ± 773.63
**2LG**	15,045.36 ± 1.472.329	17,714.40 ± 1513.11	8383.01 ± 1269.33	14,721.11 ± 3523.24
**2PG**	287,635.54 ± 126360.68	251,591.30 ± 64894.94	250,790.67 ± 90116.73	294,106.10 ± 72879.22
**2OG**	20,974.23 ± 2234.46	29,883.32 ± 3591.74	9739.69 ± 1504.49	25,774.59 ± 4962.39

Data represent mean ± SEM for 7 animals per group and are expressed as pmoles/g tissue. Different superscripted letters indicate statistically significant differences (*p* < 0.05) (one-way ANOVA nonparametric measures with Kruskal–Wallis test). AEA, *N*-arachidonoylethanolamide or anandamide; DHEA, *N*-docosahexaenoylethanolamide; DTEA, Docosatetraenoylethanolamide; LEA, *N*-linoleoylethanolamide; OEA, *N*-oleoylethanolamide; PEA, *N*-palmitoylethanolamine; POEA, palmitoleylethanolamide; SEA, *N*-stearoylethanolamide; 2AG, 2-arachidonoyl glycerol; 2LG, 2-linoleoyl glycerol, 2PG, 2-palmitoyl glycerol; 2OG, 2-oleoyl glycerol. Different letters (a,b) next to values indicate statistically significant differences (*p* < 0.05).

## Data Availability

Data is contained within this article.
